# Somatic determinants of changes in selected body posture parameters in younger school-age children

**DOI:** 10.7717/peerj.10821

**Published:** 2021-02-10

**Authors:** Marta Kinga Labecka, Krystyna Górniak, Małgorzata Lichota

**Affiliations:** 1 Department of Rehabilitation, Jozef Pilsudski University of Physical Education in Warsaw, Warsaw, Poland; 2 Department of Physical Education and Health, Jozef Pilsudski University of Physical Education in Warsaw, Branch in Biala Podlaska, Biala Podlaska, Poland

**Keywords:** Body posture, Somatic parameters, Changes, Children, Photogrammetric method

## Abstract

**Background:**

The aim of this study was to describe changes in selected parameters of body posture in children between 5 and 9 years old with diversified somatic structures.

**Methods:**

The study was carried out in 2015 and then repeated in 2018 among 67 participants who had previously been observed to have scoliotic posture. Basic body weight and height measurements were taken, which were then used to calculate the body mass index. Posture tests were conducted using the photogrammetric method.

**Results:**

Girls and boys were not significantly different in body dimensions. With age, the number of overweight boys and children with normal growth-weight proportions increased. Temporary differences in posture variables indicating abnormalities were small.

**Conclusions:**

There were no significant differences in somatic parameters between the girls and the boys. Those children with a slender body structure had the most abnormalities in the coronal plane. On the other hand, changes in spinal position in the sagittal plane were more frequent in overweight children. Relations were noted between the compensation index in the sagittal plane and deviation of the trunk inclination, the maximum deviation of the line of the spinous processes, and the angle of the shoulder line in the coronal plane and body mass index values were noted.

## Introduction

Body posture is an individual feature which forms and changes throughout a person’s life. It is described in three planes: coronal, sagittal, and transverse. Errors in body posture may include incorrect positioning of the head, shoulders, shoulder blades, trunk-arm triangles, pelvis, spine, chest, or lower limbs. Disorders in the position of specific parts of the body cause instability and imbalance  (Claus et al., 2007). To confirm this thesis and shows the abnormalities that arise, the following study describes changes in fourteen parameters of body posture in the frontal and sagittal planes. All abnormal changes in body posture intensify in periods defined as critical during the posturogenesis process. This is the beginning of junior school age and the first phase of puberty (Lafond et al., 2007). Therefore, this study covers children aged 5–9 years as the group most vulnerable to changes in body posture. Up to the age of 5, no large or sudden weight or height gains are observed. The pace of developmental phenomena at that time decrease in comparison to the period of childhood.

Between the age of 6 and 8 there is an acceleration in growth, with larger gains in body length than in body weight. During this time, the lower limbs grow rapidly, exceeding half of their body length. The physiological curves of the spine are still forming. The development of the locomotor system and changes in body proportions take place (Domljan, Vlaovic & Grbac, 2010). At the age of 7 (in a period of quite intensive developmental changes), the child starts school, which involves a lifestyle change and additional challenges. Starting school education is connected with the limitation of a child’s natural mobility, long hours of sedentary work, and stress resulting from having new experiences. The conditions are favorable to abnormal postural habits and postural defects (Hagner, Bak & Hagner-Derengowska, 2011). Apart from genetic conditions, posture is also influenced by both endogenous and exogenous factors (Modrzejewska & Malec, 2017). Many studies have indicated the relationship between body weight, height, visual and vestibular control, load distribution, postural stability, physical activity or individual shaping of body posture (De Espírito Santo, Guimaraes & Galera, 2011; Drzal-Grabiec et al., 2012; Gong et al., 2019; Hawke, Rome & Evans, 2016; Sedrez & Rosa, 2015; Leal et al., 2006; Meliscki, Monteiro & Giglio, 2011; Nery et al., 2010; Watanabe et al., 2017; Alotaibi et al., 2016; Wojtkow, Szkoda-Poliszuk & Szotek, 2018; Plandowska, Lichota & Gorniak, 2019; Walicka-Cuprys et al., 2013). A study of the relations between the factors mentioned above has allowed an identification of certain tendencies for the occurrence of abnormalities in a child’s motor apparatus. It was found that, with age, children develop relationships between their somatotype, lifestyle, physical activity or postural habits, and body posture quality. Also, Alotaibi et al. (2016) observed the effects of an absence of vision on body posture. Blind people exhibit consistent musculoskeletal deformities. In blind people, exercise should restore the normal mechanics of posture and gait. Wojtkow, Szkoda-Poliszuk & Szotek (2018) found a moderate correlation between body posture and foot load distribution in children. Hawke, Rome & Evans (2016) observed agreement between foot posture and whole-body flexibility. However, Plandowska, Lichota & Gorniak (2019) observed differences in posture stability in 5-year-old children depending on their body height. An analysis of the parameters describing posture stability varied between girls and boys. Grabara, Bieniec & Nawrocka (2017) found dependencies between the shape of lumbar lordosis and with age and gender and poor correlations between sagittal spinal curvatures and somatic parameters (body mass index). Also, Wyszynska et al. (2016) showed that the content of muscle tissue, adipose tissue and physical activity level determine the variability of the parameter characterizing the body posture. The following article describes many parameters of body posture in terms of age, sex, and somatic development in younger school-age children.

Many publications also discuss the etiology and epidemiology of posture defects in the population in the period of progressive development  (Czaprowski et al., 2018; Hagner, Bak & Hagner-Derengowska, 2011; Sedrez & Rosa, 2015). Hagner, Bak & Hagner-Derengowska (2011), examining a group of children between 6 and 10 years of age, observed a tendency for postural deterioration to increase as the children became older. The percentage of correct posture in 10-year-old children almost halved compared to those who were 6 years old, from 42% to 27%. Significan changes were observed in shoulder inclination in the sagittal plane, shoulder blade position, asymmetry of the back shape in the bend forward. Sedrez & Rosa (2015) observed that the frequency of postural changes in the studied group was 79%, 47% of which concerned changes in the coronal plane. In all the studies, there was a high percentage of asymmetries and anomalies in the positioning of particular body parts. The authors, referring to the progression of changes in body posture in the coronal plane, emphasize the importance of early diagnosis in order to catch early body statics disorders and prevent the development of scoliosis (Nissinen, Heliovaara & Seitsamo, 2000). Duval-Beaupere et al. (1970) began to analyze the development of scoliosis in the area of child development in the seventies. It can appear as a birth defect. Also, numerous internal and external factors influence the formation and development of scoliosis (Negrini et al., 2012; Vasiliadis, Grivas & Kaspiris, 2009). Scoliosis is a disadvantage that progresses quickly. Early forms of scoliosis, if not monitored, can turn into serious defects and cause disorders of the body’s functioning. That is why it is so important to monitor changes in the structure and position of the child’s body at every stage of their development and to take appropriate prophylactic and corrective measures at an early stage (Angarita-Fonseca et al., 2019; Calvo Munoz, Gomez Conesa & Sanchez Meca, 2013). Moreover serious abnormalities may cause significant deformities in the osteoarticular system, pain, and internal organ disorders, and thus significantly influence the quality of life. The high frequency of abnormalities in body posture serious health consequences in younger school-age children oblige to continue research in this direction.

The value of the research presented in this paper consists in the verification of observations concerning the 4-year period of body posture development (from 5 to 9 years old) in relation to somatic changes in children with previously diagnosed spinal changes in the coronal plane. During this time, children undergo a difficult developmental period and at the same time are subject to all the challenges resulting from starting school education. The issue in question is important, as demonstrated by the numerous studies on physical development and body posture in the initial period of junior school age. However, it should be noted that most previous studies present the results of single research on the body development and posture of children and youths.

The following studies shows to what extent these changes threaten the child’s health and development. Moreover, presents the characteristics of these changes in the area of child development.

The aim of this study was to characterize changes in selected parameters of body posture in children between 5 and 9 years old with diversified somatic structures.

The following research questions were posed:

1. How big were the changes in somatic structure and body posture occurred girls and boys between the ages of 5–9?
2. To what extent did the parameters being tested concern the position of the spine and torso related to the somatic structure of children?

## Materials & Methods

### Study design

The study was conducted for the first time in 2015 as part of project DS. 246 “The psychophysical condition of Biala Podlaska five-year-olds”, and then in 2018 as part of research project Dm. 74 “Changes in body posture of children between 5 and 9”.

### Human subjects approval statement

Both projects received a positive reaction from the Faculty and Senate Committee on the Ethics of Scientific Research at Jozef Pilsudski Academy of Physical Education in Warsaw (DM. 74–SKE 01 30/2018; DS. 246–SKE 01-01/2014). All procedures performed in the studies which involved human participants were in accordance with the ethical standards of the institutional and national research committee and with the 1964 Helsinki declaration and its later amendments or comparable ethical standards.

This study involved no danger or harm to participants.

### Written informed consent to participate

Prior to the study, parents or legal guardians gave their written consent for the child to participate in the observations and use the results for scientific purposes. Everyone was informed about the aims, methodology, and methods of conducting the research. The children were brought to the study by the parent or legal guardian. An adult was in the room during posture observation.

Informed consent was obtained from all individual participants included in the study.

### Participation criteria

**Figure 1:**
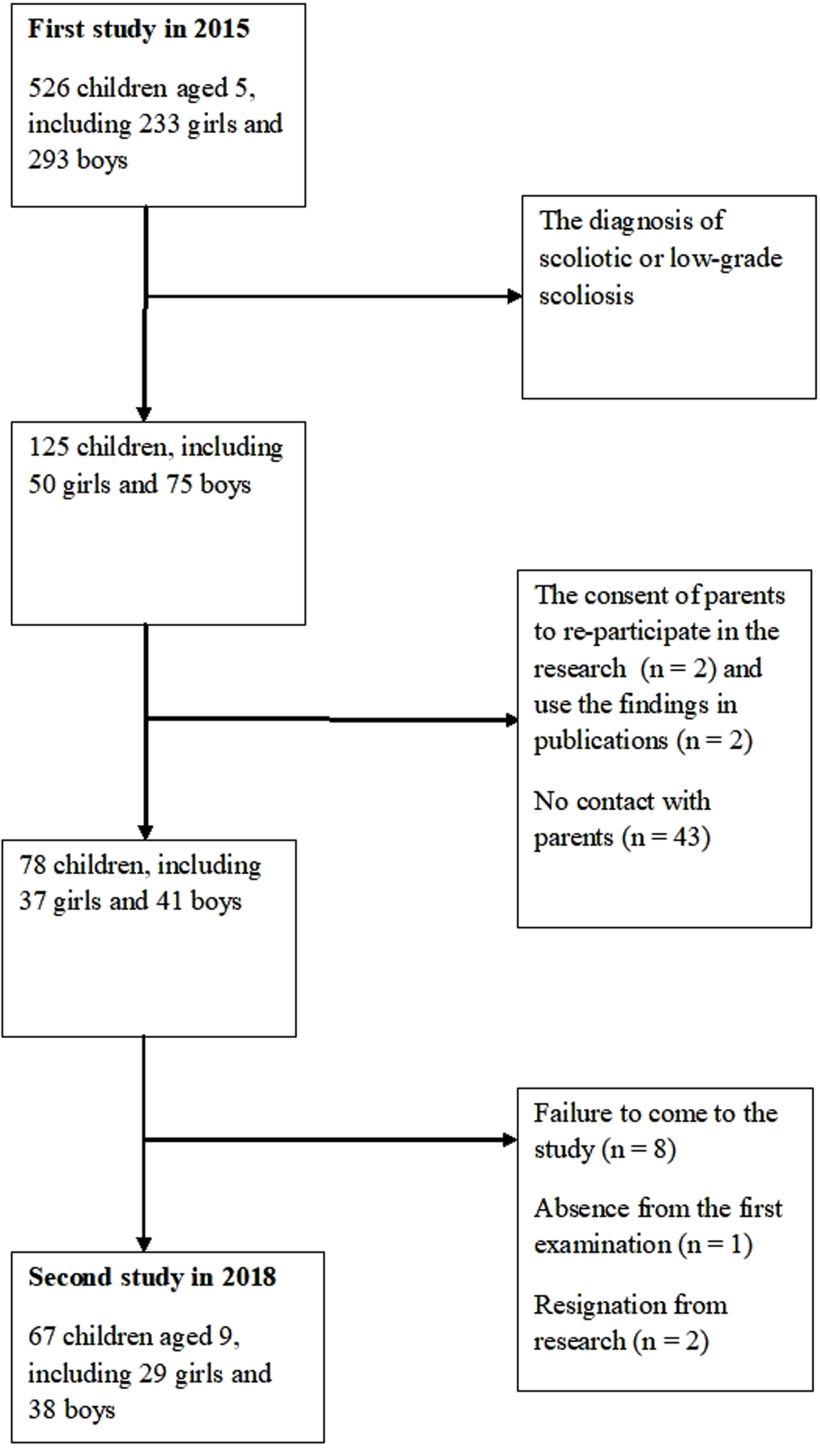
Flow chart of participants.

This paper presents the results of two studies of children in the early school period. The first study on body development and posture was conducted in 2015. They covered 526 children aged 5, including 233 girls and 293 boys. Figure 1 presented a flow chart of participants.

The following criteria were used as the inclusion criterion:

- a child born in 2010,

- enrolled in pre-school education in the city,

- parents or legal guardians consented to participate in the research.

Sick children and children living outside the city did not participate in the project.

The study indicated the occurrence of body symmetry disorders in 25% of children, and in 2018 research was organized for this group. As a result, 9-year-olds participated in the research, including 29 girls and 38 boys. The following criteria were used as inclusion criteria: the child’s participation in the research in 2015 and the diagnosis of scoliotic or low-grade scoliosis, as well as the consent of parents to re-participate in the research and use the findings in publications. The exclusion criterion was poor health.

### Methods

The evaluation of the somatic structure took into account the results of basic anthropometric parameters, such as body weight and height, which were measured on a calibrated electronic scale with a stadiometer (RADWAG, Zyrardow, Poland), with an accuracy of 0.1 kg and 0.1 cm. The obtained data were used to calculate body mass index (BMI) (WHO, 1970).

Posture tests were based on the objective method. A computer spinal and torso posture assessment was performed using MORA 4 Generation, CQ Electronic System (Swierc, 2011). In 1970 Takasaki (Takasaki, 1970) proposed Moire’s topography (MT) for analyzing body posture. It is an easy, non invasive technique of testing body posture and is suitable for use in schools and health care units. MT doesn’t not replace X-ray but may be an alternative method of examining body posture (Porto et al., 2010; Silva et al., 2014). It is known and used mainly in Japan (the most advanced country in using this method), Poland, Brazil, Iran and eastern European countries (Bosnia and Herzegovina, Serbia) (Grabara, Bieniec & Nawrocka, 2017; Silva et al., 2014; Kuroki et al., 2018; Taheri Tizabi et al., 2012; Kapo et al., 2018). Moreover, it is a reliable and repeatable method of posture evaluation (Filho et al., 2017).

### Body posture tests

The values of the measured somatic parameters of the examined children were compared to the growth charts and limit values for underweight, overweight, and obesity formulated by the World Health Organization (WHO) (WHO, 2006). On this basis, three groups of children were distinguished:

Group I—children with a stout body structure, whose weight and height, and BMI measurement values were below the 15th percentile.

Group II—children with normal growth-weight proportions who, according to WHO standards, had correct body dimensions between the 15th and 85th percentile.

Group III—children with a slender body structure, with high values of parameters, above the 85th percentile.

In these groups, changes in the analyzed parameters of body posture were presented. Body posture was assessed in accordance with the device manual (Swierc, 2011). On the child’s body, bone points were marked: spinous processes of the spine from C7 to L5, lower angles of the shoulder blades, shoulder processes, upper posterior iliac spines. Then the child assumed a standing, free, unforced position, with his back to the camera at a distance of 2.6 m. The computer program took several photos of the silhouette of the child using a photogrammetric method, using the phenomenon of Projection Moire. After selecting the appropriate photographs, linear and angular values were determined defining the position of the spine and torso only in the sagittal and coronal plane  (Swierc, 2011).

For the purpose of this work, the following spinal and torso posture parameters in the sagittal plane were analyzed: ALFA—lumbosacral spine region inclination [°], BETA—thoracolumbar spine region inclination [°], GAMMA—upper thoracic spine region inclination [°], KKP—kyphosis angle, KKP = 180 − (BETA + GAMMA) [°], KLL—lordosis angle, KLL = 180 − (ALFA + BETA) [°], MI—compensation index, KPT—the angle of the body bent to the forward/backward [°]. In the coronal plane, however, the following indicators were presented: KNT—the angle of the body bent to the side [°], UK—the maximum deviation of the line of the spinous processes from the C1-S1 line on the *x*-axis [mm], KNM—pelvic inclination angle [mm], KLB—angle of the shoulder line [mm], TT—angle of waist triangle [mm], UL—angle of scapulae line [mm], POTSI—posterior trunk symmetry index (Swierc, 2011). The POTSI indicator evaluates asymmetries in the frontal plane.

This has been described in detail by Inami et al. (1999) and  Suzuki et al. (1999). Frontal plane asymmetry indices were evaluated based on the location of the following parameters: C7 (FAI-C7), armpits (FAI-A) and waist (FAI-T). Height difference indices include levels of shoulder (HDI-S), armpits (HDI-A), and waist (HDI-T). The method of determining the individual components of the POTSI coefficient is the sum of the individual components, i.e., FAI-C7 + FAI-A + FAI-T + HDI-S + HDI-A + HDI-T (Inami et al., 1999; Suzuki et al., 1999).

The sign (-) for average values indicates the direction of changes in the spine and trunk position to the left (in the coronal plane) and the front inclination in the sagittal plane. The sign (+) indicates the direction of changes in the spine and trunk position to the right and the backward. All the devices listed and the test methods and techniques used were non-invasive.

### Statistical analysis

The Kolmogorov–Smirnov test was used to check the distribution normality of the analyzed variables. The test was also used in this study because it is intended for a study in which the test group consists of more than 50 people. The analysis of variance (ANOVA) in a one-way scheme checks whether one independent variable (factor) affects the results of one dependent variable. It is used, for example when the independent variable has a minimum of 3 or more levels (for example when comparing the change in parameters over time). In addition, it is used as one of the methods for comparing means across multiple groups or across multiple variables. The latter use is considered repeated measures ANOVA, because we are comparing repeated measures simultaneously on the same object (or observation). Analysis of variance in an intergroup design is used to analyze several intragroup factors simultaneously, taking into account the effect of interactions between them. A significance level of *p* < 0.05 was adopted (Lyman Ott & Longnecker, 2015). IBM SPSS STATISTICS (version 24.0, IBM Poland Co., Warsaw, Poland) was used to run the data/statistical analysis.

## Results

The characteristics of the somatic parameters of the examined girls and boys are presented in Table 1. The girls aged five were slightly heavier and taller than the boys. At the age of nine, however, the boys were characterized by larger body dimensions. The differences between the sexes that were observed in somatic parameters were not statistically significant (*p* > 0.05). On the cusp of four years, the body dimensions increased: body weight by 9.22 kg (girls) and 11.60 kg (boys), body height by 19.72 cm, and 21.5 cm respectively. The average BMI values were similar in the girls and the boys in the age groups analyzed (Table 1).

**Table 1:** Characteristics of somatic parameters in tested children.

Parameters	I examination	II examination	*P*
	$\overline{x}$± SD	$\overline{x}$± SD	
	Girls		
Body weight [kg]	20.14 ± 3.60	29.36 ± 6.28	0.574
Body height [cm]	114.62 ± 5.47	134.34 ± 6.79	0.571
BMI [kg/m^2^]	15.20 ± 1.62	16.10 ± 2.12	0.477
	Boys		
Body weight [kg]	19.47 ± 2.04	31.07 ± 4.70	0.159
Body height [cm]	114.55 ± 3.91	136.05 ± 5.04	0.178
BMI [kg/m^2^]	15.02 ± 1.05	16.72 ± 1.83	0.362

**Notes.**

Body mass index (BMI), ANOVA analysis (* *p* < 0.05).

Table 2 presents data illustrating the qualitative assessment of the somatic structure of the children examined. On the cusp of four years, the somatic structure increased: normal height-weight proportions in girls and decreased: excessive body weight in the girls and boys, overweight in girls, and a slim body in all children.

**Table 2:** Qualitative assessment of the somatic structure of the children examined.

Group deviation	BMI [kg/m^2^]											
	Girls				Boys				Total			
	I examination		II examination		I examination		II examination		I examination		II examination	
	[n]	[%]	[n]	[%]	[n]	[%]	[n]	[%]	[n]	[%]	[n]	[%]
Group I	9	31.0	5	17.2	14	36.8	3	8.0	23	34.3	8	11.9
Group II	15	51.8	22	76.0	22	57.9	23	60.5	37	55.2	45	67.2
Group III	5	17.2	2	6.8	2	5.3	12	31.5	7	10.4	14	20.1

**Notes.**

Number of persons [n], frequency of parameters occurrence among children [%], body mass index (BMI).

The area of interest was the characteristics of basic body posture parameters in children with different somatic conditions. The differences between the measured parameters are presented in Table 3. In the children aged 5 with normal height-weight proportions were observed: the predominance of thoracic kyphosis over lumbar lordosis, a maximum torso backward inclination angle, the right iliac spike, and the lower shoulder blade angle were higher set than the left. In children with a slender body the greatest differences were observed in the waist triangle height and the flatter curves of the spine. In children with overweight was observed: the torso left inclination angle, the maximum deviation of spinous processes lines from C7-S1 in the coronal plane to the left and left shoulder was higher than the right one.

**Table 3:** Differences in body posture parameters depending on body mass index (BMI) between I and II examination.

Parameters	Examination	Total	*p*	Group I	Group II	Group III
		$\overline{x}$± SD		$\overline{x}$± SD	$\overline{x}$± SD	$\overline{x}$± SD
ALFA [°]	I	7.72 ± 3.59	0.122	8.32 ± 2.95	7.46 ± 4.07	7.13 ± 2.88
	II	6.76 ± 4.30		6.69 ± 4.63	6.92 ± 4.62	6.31 ± 3.15
BETA [°]	I	8.15 ± 3.59	0.702	8.31 ± 3.66	7.82 ± 3.25	9.33 ± 5.15
	II	8.02 ± 3.04		8.49 ± 3.41	7.87 ± 3.18	8.28 ± 2.53
GAMMA [°]	I	9.45 ± 3.75	0.500	9.28 ± 4.11	9.66 ± 3.14	8.90 ± 5.73
	II	9.80 ± 3.41		9.28 ± 3.78	9.91 ± 3.61	9.74 ± 2.67
KKP [°]	I	162.78 ± 6.54	0.634	162.40 ± 6.36	163.02 ± 6.05	162.74 ± 10.04
	II	162.20 ± 5.59		162.96 ± 4.99	162.08 ± 6.08	162.16 ± 4.40
KLL [°]	I	164.41 ± 6.17	0.392	163.36 ± 4.67	165.22 ± 6.90	163.53 ± 6.71
	II	165. 00 ± 6.87		162.45 ± 9.98	165.42 ± 7.09	165.40 ± 3.34
**MI**	I	**1.68 ± 5.98**	**0.07**	0.95 ± 5.83	2.20 ± 5.73	0.78 ± 8.17
	II	**4.26 ± 7.15**		4.97 ± 8.81	4.21 ± 7.65	3.99 ± 4.32
KPT [°]	I	−0.88 ± 2.75	0.609	−0.85 ± 2.64	−1.10 ± 2.58	0.24 ± 3.95
	II	−0.82 ± 2.54		−0.98 ± 2.56	−0.53 ± 2.64	−1.65 ± 2.09
**KNT** [°]	I	**-0.73 ± 1.28**	**0.002**	−0.63 ± 1.21	−0.72 ± 1.32	−1.10 ± 1.48
	II	**-0.01 ± 1.27**		0.24 ± 1.03	−0.06 ± 1.34	0.05 ± 1.28
**UK** [°]	I	**-0.63 ± 2.17**	**0.000**	−3.05 ± 5.15	−3.45 ± 4.28	−5.27 ± 2.54
	II	**-3.50 ± 4.45**		0.50 ± 2.14	−0.86 ± 2.23	−0.52 ± 1.89
KNM [mm]	I	0.35 ± 8.58	0.995	−0.05 ± 3.07	0.56 ± 2.57	0.41 ± 2.98
	II	0.27 ± 7.46		1.36 ± 1.94	0.18 ± 3.18	−0.05 ± 1.17
**KLB** [°]	I	**3.63 ± 7.46**	**0.01**	1.25 ± 7.93	−0.35 ± 8.76	−1.84 ± 10.45
	II	**0.04 ± 8.58**		4.66 ± 8.40	3.19 ± 7.67	4.47 ± 6.59
TT [mm]	I	0.93 ± 10.05	0.796	2.55 ± 9.56	0.20 ± 10.21	1.86 ± 5.88
	II	1.18 ± 9.58		2.54 ± 12.38	0.63 ± 10.42	1.00 ± 7.79
UL [mm]	I	1.18 ± 9.58	0.06	0.71 ± 5.67	0.79 ± 7.67	−0.34 ± 4.60
	II	0.93 ± 10.05		3.46 ± 6.87	3.14 ± 6.13	1.03 ± 7.05
POTSI	I	19.46 ± 9.59	0.911	18.79 ± 7.37	19.44 ± 10.51	21.22 ± 7.50
	II	19.22 ± 9.25		14.50 ± 5.92	20.21 ± 9.15	18.71 ± 10.71

**Notes.**

Average ($\overline{x}$) standard deviation (SD) ALFA lumbosacral spine region inclination [°] BETA thoracolumbar spine region inclination [°] GAMMA upper thoracic spine region inclination [°] KKP kyphosis angle KKP 180 − (BETA + GAMMA) [°] KLL lordosis angle KLL 180 − (ALFA + BETA) [°], MI compensation index KPT the angle of the body bent to the forward/backward [°] KNT the angle of the body bent to the side [°] UK the maximum deviation of the line of the spinous processes from the C1–S1 line on the *x*-axis [mm] KNM pelvic inclination angle [mm] KLB angle of the shoulder line [mm] TT angle of waist triangle [mm] UL angle of scapulae line [mm] POTSI posterior trunk symmetry index

ANOVA analysis: statistically significant results between I and II examination (*p* < 0.05).

In the children aged nine with a slender body structure, the most changes were observed: the largest trunk inclination to backward and right, increase the asymmetry of the shoulders, scapulas and pelvis, the greatest differences in waist triangle height (Table 3).

In children examined, on the cusp of four years , the parameters increased: MI (*p* = 0.07), and UK (*p* > 0.001) and decreased: KNT (*p* = 0.002), and KLB (*p* = 0.01).

Table 4 shows differences in body posture parameters between the groups of children examined. Statistically significant differences only occurred in the parameters UL (*p* = 0.04) and UK (*p* = 0.04) between the group of children with stout and slender body structure (Table 4).

**Table 4:** Differences in body posture parameters between the groups of children examined.

Parameters	Examination	Group I–II	Group I–III	Group II–III
		*p*	*p*	*p*
ALFA [°]	I	0.382	0.353	0.838
	II	0.895	0.824	0.648
BETA [°]	I	0.389	0.563	0.313
	II	0.619	0.871	0.663
GAMMA [°]	I	0.688	0.847	0.615
	II	0.650	0.737	0.872
KKP [°]	I	0.708	0.915	0.921
	II	0.721	0.698	0.991
KLL [°]	I	0.258	0.940	0.553
	II	0.285	0.317	0.924
MI	I	0.418	0.952	0.578
	II	0.801	0.727	0.917
KPT [°]	I	0.720	0.399	0.253
	II	0.655	0.517	0.154
KNT [°]	I	0.810	0.403	0.491
	II	0.546	0.727	0.778
UK [°]	I	0.213	**0.04**	0.308
	II	0.117	0.258	0.611
KNM [mm]	I	0.444	0.752	0.707
	II	0.894	0.440	0.280
KLB [°]	I	0.690	0.839	0.443
	II	0.623	0.953	0.572
TT [mm]	I	0.863	0.780	0.566
	II	0.644	0.722	0.902
UL [mm]	I	0.968	0.659	0.892
	II	0.314	**0.04**	0.796
POTSI	I	0.795	0.432	0.607
	II	0.09	0.320	0.609

**Notes.**

ALFA lumbosacral spine region inclination [°] BETA thoracolumbar spine region inclination [°] GAMMA upper thoracic spine region inclination [°] KKP kyphosis angle KKP 180 − (BETA + GAMMA) [°] KLL lordosis angle KLL 180 − (ALFA + BETA) [°] MI compensation index KPT the angle of the body bent to the forward/backward [°] KNT the angle of the body bent to the side [°] UK the maximum deviation of the line of the spinous processes from the C1–S1 line on the *x*-axis [mm] KNM pelvic inclination angle [mm] KLB angle of the shoulder line [mm] TT angle of waist triangle [mm] UL angle of scapulae line [mm] POTSI posterior trunk symmetry index

ANOVA analysis: statistically significant results between groups (*p* < 0.05).

## Discussion

The aim of this study was to characterize changes in selected parameters of body posture in children between five and nine years old with diversified somatic structures. The authors of the study made a strong attempt to achieve this aim, thus proving the occurrence of changes in somatic parameters and body posture parameters in junior school-age children.

The literature shows that the early school period is difficult in terms of the process of posturogenesis. During this period, children are most exposed to changes in body posture. Approximately 50% of children exhibit changes in posture in the frontal plane (Hagner, Bak & Hagner-Derengowska, 2011; Sedrez & Rosa, 2015; Czaprowski et al., 2018), and 2–3% have idiopathic scoliosis (Angarita-Fonseca et al., 2019). Moreover, the findings of initial studies showed an unfavorable shaping of the child’s locomotor system. One in four five-year-olds was characterized by body posture disorders. Situations showing the extent to which changes occur or may occur in children provided a justification for taking up the topic.

Our research opines, in the group of the examined children, both at the age of five and nine, no clear sex differentiation of somatic parameters was observed, although the girls aged five were slightly heavier and taller than the boys. On the other hand, at the age of nine, it was the boys who had higher body weights and heights than the girls. Different results were obtained by Twardus, Kozka & Struska-Depowska (2011). In their studies show that in the group of boys, the analysis of the obtained results of body height measurements in relation to the percentile norms indicated low height in three subjects, weight deficiency in one and excess body weight in three. In the group of girls, short stature was found in three of them (8.1%), weight deficiency in three and excess body in three subjects. The results our research are partly compatible with studies Perenc, Radochonska & Blajda (2016). The authors was observed first jump body height occurred in boys aged five-six years, and in girls a year later. Between the ages of four and eighteen, both genders tend to increase systematically in height. Nine-year-old boys experienced a significant increase in body weight. Dimeglio (2001) concluded that all changes in body length dimensions are gradual and each dimension has its own period of rapid growth.

By analyzing the BMI values from pre-school to early school, girls tend to slenderness while boys stout body built. Our research shows that the majority of boys had normal growth-weight proportions compared to girls. The only disturbing situation was the increase in the number of overweight cases in nine-year-old boys. The study of six to seven-year-old children conducted by Twardus, Kozka & Struska-Depowska (2011) showed that only 1/5 of the respondents had abnormal BMI values. The vast majority of boys (78.8%) and girls (75.5%) were normal weight and height parameters. Other studies show that among pre-school children, the prevalence of overweight was 9.1% girls and 9.9% boys, and obesity in 7.2% girls and 8.4% boys. Repetition of the research in early school age showed that obesity among girls significantly decreased (10% vs 7.7%), while in boys its incidence stabilized (6.8% vs 6.4%) (Kedzior et al., 2017).

The above aspects are relevant because proper development is one of the main factors determining the ability to meet new requirements and obligations faced by a child at school. It is assumed that to start schooling, a mature child should be sufficiently physically developed (level of construction and functions of systems and organs, which provides resistance to fatigue, disease) and have the appropriate motor skills.

Research by other authors shows that somatic structure differentiates body posture. A large proportion of body posture defects occur in children who are underweight and obese. In children with a slender body, scoliotic posture and scoliosis are more frequently observed, while in overweight and obese children, changes in the physiological curves of the spine are more common (Gorniak et al., 2014; Burdukiewicz, Milkowska & Pietraszewska, 2006; Trzeciak & Barczyk-Pawelec, 2014; Wyszynska et al., 2016). Our studies conducted showed very clear relationships between the somatic structure and the body posture quality of girls and boys. Children with low scoliosis were slimmer compared to their peers who had normal posture (Gorniak et al., 2014). Research by Wyszynska et al. (2016) showed that overweight children had a reduced angle of the thoracolumbar section inclination. Burdukiewicz, Milkowska & Pietraszewska (2006) and Trzeciak & Barczyk-Pawelec (2014) observed that changes in the anterior-posterior curvature of the spine are more frequent in children with a slender body structure.

Our study also shows that over the years, in overweight children, spinous processes could significantly reduce their distance from the C7-S1 line to the left. In children, significant differences in average values occurred in the case of KNT, MI, UK and KLB parameters. Drzal-Grabiec et al. (2012) examined a group of two hundred and ninety-three children aged seven-nine, using the photogrammetric method. They observed different results. In the examined children, there were significant differences in UL and KNM parameters. However, Nery et al. (2010), while examining Brazilian children, showed a relationship between abnormal shoulder position (KLB) and shoulder blades (UL) and body structure. The lack of connection between BMI and the occurrence of posture defects was pointed out by Latalski et al. (2013) and Fu et al. (2014).

Based on the data provided in this study, the authors can therefore recommend that there is need for a child’s posture to be regularly monitored, and where possible, controlled. This recommendation should be welcomed especially in the case of overweight or underweight children. Research shows that these are the groups most prone to posture abnormalities. All the burdens resulting from compulsory education also contribute to the formation of posture defects.

## Conclusions

Differences in the average values of the spinal and torso posture variables measured between the groups of children were small and not statistically significant. It was also found that those children with scoliosis or low scoliosis with a slender body structure had the most abnormalities in the coronal plane. On the other hand, the predominance of kyphosis over lumbar lordosis, and a decrease in the backward torso inclination angle were more frequent in the overweight children.

Children with a slender body are more predisposed to the progression of changes in the lateral curvature of the spine. This research shows the importance of monitoring a child’s body posture in terms of developmental changes and lifestyle in prophylactic health care for children and adolescents. A recommendation for future research may be to assess the children again at puberty and afterward. Continued observation may indicate changes/direction of changes in body posture related to the quality of somatic development.

##  Supplemental Information

10.7717/peerj.10821/supp-1 Data S1 Measured parameters in children

## Additional Information and Declarations

### Data Availability

The following information was supplied regarding data availability: Raw data are available as Supplemental File.
